# Supplementary material for: Aberrant regulation of a poison exon caused by a non-coding variant in a mouse model of Scn1a-associated epileptic encephalopathy
Source: PLoS Genet. 2021 Jan 7;17(1):e1009195. doi: 10.1371/journal.pgen.1009195 (PMC7790302; doi:10.1371/journal.pgen.1009195)
Supplement: Attachment — Submitted filename: ReplyToReviewers.docx [file pgen.1009195.s007.docx]

We thank the reviewers for their positive feedback on our manuscript. We were especially glad to hear that the “**work is very detailed and provides a good rationale for this new genetic mechanism**” and “**the results are clearly presented, and the manuscript is well written**.” We have addressed all points raised by reviewers in the revised manuscript as described below. All changes are highlighted in red in the manuscript.

Reviewer #1: Voskobiynyk et al present their findings in support of a "poison exon" model for some patients with Dravet syndrome and show that an intron 20 mutation found in patients recapitulates a Dravet syndrome-like phenotype in a CRISPR/Cas9 knockin mouse model. Overall, their data are compelling that the intronic mutation c.3969+2451G>C leads essentially to a haploinsufficiency state for SCN1A. They argue, though do not prove, that this is due to nonsense-mediated decay. Building on previous work with a poison exon in SCN8A, they also show that the poison exon is expressed during fetal development, and only becomes a minor species postnatally.

1. It would be helpful if the authors might briefly expand on the time points where exon 20N becomes a minor species (P15), and when the Dravet phenotype emerges. Is the increased death in SCN1A+/KI mice seen only around P15, but not at P7, for example?

The stress of handling nursing dams and offspring makes it challenging to evaluate Dravet syndrome-like phenotypes in detail prior to weaning. Anecdotally, on several occasions we were able to count newborn litters in the nest and observed decreased offspring numbers at weaning, leading us to speculate that some offspring died prematurely before the third or fourth week of life, as in other Dravet syndrome models. These observations are noted in the revised manuscript (**lines 137–143)**. More broadly, the relationship between the time course of normal poison exon usage and seizure onset in *Scn1a* mutant mice is of course relevant to the onset of seizures in Dravet syndrome (as noted by Reviewer 2, point #6). In the revised manuscript, we have discussed this issue in the context of recent data on human *SCN1A* expression and speculate that our observations provide a likely mechanistic explanation for the time at which Dravet syndrome seizures may first become apparent (**lines 240–249**).

2. (a) There is also some literature to suggest an association of SCN1A mutations with SUDEP and fatal cardiac arrhythmias, and possibly liver toxicity. Perhaps, although the authors find lower SCN1A expression in heart and liver, these lower expressions should not be dismissed in the pathophysiology of DS. (b) The 20N exon mRNA is undetectable in several of the non-brain tissues. Does this suggest that the poison exon does not explain reduced SCN1A expression in these tissues compared to brain?
(a) Our interpretation of the literature Nicolai et al., [1] is that liver toxicity is likely secondary to treatment rather than a primary consequence of *SCN1A* deficiency. Similarly, prior work by Kalume et al. [2] suggests that cardiac arrhythmias and SUDEP in *Scn1a* mouse models is likely secondary to brain dysfunction since a brain-specific but not a cardiac-specific KO of *Scn1a* led to lethal bradycardia. We are aware, however, of the electrophysiology work by Auerbach et al. [3] that suggests a potential primary role for *Scn1a*. In the revised manuscript, these issues have been raised briefly in the Discussion (**lines 260–262**), and the relevant references added.
(b) We have been unable to detect exon 20N expression in any tissue other than the brain, despite exhaustive attempts to do so (multiple nested primer sets, analysis of total RNAseq datasets). This suggests that reduced *Scn1a* expression in non-neuronal tissues does not involve a poison exon mechanism and instead is more conventional, e.g. chromatin accessibility and/or transcription factor availability. This point has been added to the Discussion (**lines 250–260**).
3. The discussion of targeting exon 20N with therapeutics to treat DS is intriguing, though the mechanism of exon 20 formation likely needs to be clarified. This work would certainly be strengthened by mechanistic data regarding the incorporation of exon 20 in normal development and the increased incorporation in patients with the c.3969+2451G>C mutation. However, I do not think this additional mechanistic data is essential for the authors to argue their case in this study.

In the revised manuscript, we have added a brief discussion of the potential underlying mechanisms for exon 20N inclusion (**lines 250–255**).

4. A minor point: SMA pathophysiology is not explained correctly in the discussion: SMA is not caused by haploinsufficiency of SMN2, but by homozygous loss of SMN1, with copy number of SMN2 influencing severity and onset of disease. It is true that nusinersen, an antisense oligonucleotide, is used to treat SMA by modifying splicing (incorporation of exon 7). This should be corrected in the manuscript, along with a few grammatical errors. Overall, the manuscript is clear and well-written.

We have corrected this point in the revised manuscript to refer broadly to the use of oligonucleotide-based therapies to modulate alternative splicing (in DMD as well as in SMA) (**lines 268–272**).

Reviewer #2: Voskobiynyk and collaborators report on the functional effects of a "poison exon" variant in SCN1A, a recently identified genetic mechanism in Dravet Syndrome. The authors report on the effect on a mouse model, replicating the phenotype and provide a conceptual framework on how a deep intronic variant affects poison exon inclusion and gene regulation. Their work is very detailed and provides a good rationale for this new genetic mechanism. I have the following comments and questions.

1. Seizure phenotype of SCN1A+/KI mice. The authors provide relatively little information on the seizure phenotypes of the SCN1A+/KI mice and do not provide some of the detailed EEG analysis that was used in other mouse models for SCN1A. Could the authors provide some more quantitative information on seizures in the SCN1A+/KI mice to make the case that their model aligns with prior SCN1A mouse models? Given the overall complexity of the manuscript, I would not require the authors to generate additional data, but simply provide existing data in a more comparative way.

In developing our research plan, we chose to pursue detailed behavioral phenotyping rather than seizure phenotyping (EEG, electrophysiology, quantitating the response to PTZ), in part because the literature is already replete with these types of studies in *Scn1a* mutant mice (but less so with behavioral studies), and in part because our results on RNA and protein expression make a compelling case that the point mutation we introduced leads to a loss of Na_v_1.1 protein. Anecdotally, we or the animal caretakers frequently observed generalized seizures in the cohort, but quantitative records are unfortunately not available, beyond the comparative phenotypic data presented in Figures 3, S3, and S4.

2. Reference to clinical features of index patients. The authors refer to their index patient with the c.3969+2451G>C variant, but the reader is required to return to the prior publication by Carvill and collaborators to obtain more information. Could the authors provide a brief synopsis of the patient's clinical features and genetic features (de novo status of the c.3969+2451G>C variant)?

We agree that this information would increase the reader’s understanding of the background of our study and have added the synopsis of the clinical and genetic features of the patient with the c. 3969+2451G>C variant (**lines 102–105**).

3. Survival of SCN1A+/KI mice. The authors provide a sufficient description on how SCN1A+/KI mice replicate prior Dravet Syndrome mouse models. However, following the description alone is relative difficult at times. Would it be possible for the authors to add information on "historic comparisons" for other DS models to Figure 3 (or some parts of Figure 3), for example by adding another survival curve in Figure A to compare SCN1A+/KI mice to prior DS models?

It would be challenging to add survival curves based on other published data to Figure 3; equally important, survival of hemizygous animals depends on genetic background. However, we have revised and expanded the relevant section of the Results (**lines 140–143**) to provide qualitative comparisons of survival between our *Scn1a* +/*KI* animals and prior work, and to ensure that this data is clear and accessible to the reader.

4. (a) Ratio of inclusion of poison exon. In lines 171-174, the authors conclude that nearly all transcripts from the allele contain exon 20N compared to only 1% in WT mice. Could the authors expand on how they arrive at this number? It becomes obvious from comparing the reduction in overall SCN1A expression, but is this the only conclusion? Could the exon 20N mutation have triggered other transcriptional events that reduce SCN1A expression? (b) Also, could the authors provide hypothesis how the increased inclusion is achieved? Is there anything known about regulatory elements in exon 20N that may have triggered increased/decreased binding of RNA-binding proteins that lead to the inclusion?
(a) Our RNA-seq data show no evidence of additional transcriptional events that might affect *Scn1a* expression; therefore, the ~50% reduction of *Scn1a* RNA in +/*KI* animals implies that nearly all of the *KI* transcription from the *KI* allele includes exon 20N, which then becomes rapidly degraded, and there are no additional conclusions. This point has been expanded upon in the revised manuscript (**lines 170–183**).
(b) Also suggested by reviewer 1 (point 3) and reviewer 3 (point 1b), we have added a brief discussion in the revised manuscript of how the underlying mechanisms of exon 20N formation and inclusion might be pursued (**lines 250–260**).
5. Developmental trajectory of poison exon inclusion. The authors should be complemented for their bioinformatic analysis of exon 20N inclusion in SCN1A and SCN8A. This data shows that 'poison exon inclusion' is not a pathological mechanism in itself, but that the disease mechanism in SCN1A and SCN8A is 'persistent poison exon inclusion', a defect of turning off a likely physiological developmental mechanism. The authors can consider whether they want to point out this aspect (optional).

In the revised manuscript, we have emphasized this point in the Discussion, lines (**lines 217–221**).

6. Mouse vs. human development. Could the authors provide references on how mouse development corresponds to human development with respect to SCN1A expression. It may be implied from their paper that physiological poison exon inclusion ends at an estimated 3-6 months age in human when clinical features of DS become apparent, but a clinical reader may not be able to infer the human age at mouse age P15.

Also suggested by reviewer 1 (point 1), we have in the revised manuscript discussed (**lines 240–249**) this issue in the context of recent data on human *SCN1A* expression and speculate that our observations provide a likely mechanistic explanation for the time at which Dravet syndrome seizures may first become apparent.

Reviewer #3: The authors developed another mouse model for Dravet syndrome (DS) based on knock-in of a variant in poison exon 20N in the gene SCN1A which they described previously in a DS patient. They nicely show that the mechanism is evolutionary conserved and that overexpression of the allele with a poison exon is triggered by the mutation and leads to a decrease of Nav1.1 channels of about 50%, comparable to other DS models. They further show that there is developmental regulation of Nav1.1 including poison exon 20N which contributes to the relatively late upregulation of Nav1.1 in development. A similar mechanism is confirmed for Nav1.6 which had been described previously. The results are clearly presented and the manuscript is well written.

Critique

Major points

1. The manuscript stays on a descriptive level and the potential gene regulatory mechanisms are neither examined nor discussed. (a) The authors describe that Nav1.1 expression in other than neuronal tissues is low throughout development, suggesting that the SCN1A promoter is responsible for tissue-specific expression of the gene and that the poison exon does not contribute to suppression of Nav1.1 expression in other tissues. The authors do not discuss this issue. (b) I am not an expert in gene regulation, but the authors mention other diseases like cancer in which poison exons play a role. Thus, it may be that mechanisms have been examined as to why poison exons are expressed at different levels under various conditions, such as a disease-causing mutation or developmental regulation. At the very least, the authors should mention known mechanisms of gene regulation by poison exons and they should discuss different roles of promoter- and poison-exon-mediated mechanisms.
(a) Also suggested by Reviewer 1 (point 2b), we have expanded the discussion in the revised manuscript to address this point (**lines 250–260**).
(b) And, as suggested by Reviewer 1 (point 3) and Reviewer 2 (4b), we have added a brief discussion in the revised manuscript of the potential role of poison exon-mediated gene regulation vs. more conventional, e.g. chromatin accessibility, transcription factor-mediated mechanisms (**lines 250–260**).

Minor points

2. Appropriate original literature should be cited on DS/SCN1A, not only reviews (refs 5,6). Claes et al. AJHG 2001, Yu et al. Nat Neurosci 2006, Ogiwara et al. J Neurosci 2007, Hedrich et al. J Neurosci 2014 are key papers with regard to genetics and pathophysiology of SCN1A defects including widespread interneuron and network dysfunction which should be cited right at the beginning.

These references were cited in the Discussion section of the original manuscript; in the revised manuscript, these references are now cited at the beginning (**lines 73–75**).

3. The authors should mention in the results section that premature death in DS mouse models largely depends on genetic background and they should report the background they used not only in the methods section.

This has been explicitly addressed in the revised manuscript with regard to both specifying the genetic background in the Results (**lines 120–123**), and in comparing our data on survival with previous work (**lines 140–143**).

4. Fig. 1D: The linkers between segments S4 and S5 have a similar length in all four domains. The figure suggests that it is longer in domain 3, which is not the case. Please adapt the figure accordingly.

We have amended Figure 1D to now show the position of poison exon 20N.

**References**

1.	Nicolai J, Gunning B, Leroy PL, Ceulemans B, Vles JSH. Acute hepatic injury in four children with Dravet syndrome: Valproic acid, topiramate or acetaminophen? Seizure. 2008;17(1):92-7. doi: <https://doi.org/10.1016/j.seizure.2007.07.003>.

2.	Kalume F, Westenbroek RE, Cheah CS, Yu FH, Oakley JC, Scheuer T, et al. Sudden unexpected death in a mouse model of Dravet syndrome. The Journal of clinical investigation. 2013;123(4):1798-808. Epub 2013/03/26. doi: 10.1172/jci66220. PubMed PMID: 23524966; PubMed Central PMCID: PMCPMC3613924.

3.	Auerbach DS, Jones J, Clawson BC, Offord J, Lenk GM, Ogiwara I, et al. Altered cardiac electrophysiology and SUDEP in a model of Dravet syndrome. PloS one. 2013;8(10):e77843. Epub 2013/10/25. doi: 10.1371/journal.pone.0077843. PubMed PMID: 24155976; PubMed Central PMCID: PMCPMC3796479.
